# Genetic association analysis between LDL-c lowering drugs and portal hypertension using Mendelian randomization analysis

**DOI:** 10.1038/s41598-025-08153-5

**Published:** 2025-07-01

**Authors:** Qing-Ao Xiao, Xiao-Long Li, Lei Qin, Xiao-Lin Zhang

**Affiliations:** 1 Department of Interventional Radiology, the First College of Clinical Medical Science, China Three Gorges University, Yichang, 443003 Hubei Province China; 2 Department of Interventional Radiology, Yichang Central People’s Hospital, Yichang, 443003 Hubei Province China

**Keywords:** Drug-target Mendelian randomization, Lipid-lowering drugs, Anticoagulation, Portal hypertension, Hepatology

## Abstract

Clinical guidelines recommend the use of statins to reduce portal pressure and alleviate portal hypertension (PH). However, there is a lack of population-level studies on the use of non-statin Low-Density Lipoprotein Cholesterol (LDL-c) reduction agents for the treatment of PH. This study utilized a novel method, Mendelian Randomization (MR) analysis, to investigate the impact of commonly used LDL-c-lowering medications on PH. Instrumental variables (IVs) for eight lipid-lowering drug-related genes were extracted from three large-scale LDL-c databases of Genome-Wide Association Studies (GWAS), followed by MR analysis. The MR results indicated that, compared to normal individuals, lower expression of CETP and NPC1L1 in whole blood (result of meta-analysis: CETP [OR: 0.322, 95%CI:0.130–0.795, *P* = 1.396e-02], NPC1L1 [OR: 0.057, 95%CI: 0.022–0.146, *P* = 2.670e-09]) is associated with reduced portal pressure. The IVs of target genes were subjected to MR analysis with coronary atherosclerosis (CAD) as a positive control, confirming that the IVs can effectively substitute for the biological function of the target gene, thereby further enhancing the reliability of the results. Subsequently, Summary-based Mendelian Randomization (SMR) analysis was conducted by using expression quantitative trait loci (eQTL) data to validate the results of the MR analysis. The SMR results suggested that only NPC1L1 is associated with PH (OR: 0.648, 95%CI: 0.472–0.891, *P*_*SMR*_ = 7.502e-3, *P*_*HEIDI*_ = 0.747) from genetic correlation. Additionally, mediation analysis indicates that the therapeutic effect of NPC1L1 inhibitors on PH is partially mediated by tissue factor (mediating effect accounted for 18.52%, and the *P* value was 0.01). Phenome-Wide MR indicated that NPC1L1 inhibitors may be associated with 23 diseases or symptoms. In addition, NPC1L1 had genetic correlation between and alkaline phosphatase as well as total bilirubin, but no genetic correlation with alanine aminotransferase, aspartate aminotransferase, direct bilirubin, or gamma glutamyltransferase. In conclusion, this study systematically analyzed the genetic correlation between lipid-lowering drug targets and PH. From a genetic correlation perspective, we revealed that the potential therapeutic effect of NPC1L1 on PH may not be mediated through the reduction of LDL-c but rather through the modulation of tissue factors. Additionally, the potential side effects associated with NPC1L1 inhibition also were explored.

**Supplementary Information:**

The online version contains supplementary material available at 10.1038/s41598-025-08153-5.

## Introduction

Portal hypertension (PH) is a major complication in the end stages of liver cirrhosis^1^. When the liver is damaged by multiple factors, regenerative nodules replace normal liver tissue, ultimately increasing intrahepatic vascular resistance and elevating portal pressure^1^. It is typically diagnosed via hepatic venous pressure gradient (HVPG), and it was appeared clinical symptoms manifesting when HVPG exceeds 10 mmHg, which was defined as clinically significant portal hypertension (CSPH)^2^. Persistent portal pressure elevation leads to symptoms such as acute gastrointestinal bleeding and refractory ascites, which may be life-threatening^2^. Gastrointestinal bleeding due to PH carries a mortality risk of up to 20% within six weeks of onset, which is further increased if accompanied by infection or other risk factors^1^. The etiology of PH is diverse. Some researchers believe that when the liver is exposed to risk factors, intrahepatic micro-thrombosis and sinusoidal fibrosis can disrupt blood flow, activate fibroblasts, and increase collagen deposition^3^. This ultimately results in hepatocyte death and fibrous septa formation, and increased intrahepatic vascular resistance, leading to impair portal blood flow and ultimately the development of PH^3^.

Previous animal study had confirmed that both short-term and long-term use of exogenous heparin significantly reduces intrahepatic vascular resistance, which was not affect arterial pressure, and inhibits liver fibrosis progression^4^. Interestingly, some lipid-lowering drugs are involved in the anticoagulation process (e.g., statins^5^, NPC1L1 inhibitors^6^) and recent guidelines also recommend the use of statins to treat PH^2^. In cirrhotic mice, acute anticoagulation did not show significant hemodynamic changes, intrahepatic resistance, however, significantly reduced after short-term or long-term anticoagulation^4^. This aligns with clinical studies of statins reducing portal pressure^7,8^.

However, some studies also displayed that statins do not reduce portal pressure. In 2023, Kronborg et al. published a randomized controlled trial (RCT) demonstrating that continuous administration of Atorvastatin (10–20 mg/day) for six months did not result in a significant difference in portal pressure compared to the control group^9^. The only differences observed between the two groups were in CD62-L-selectin, matrix metalloproteinases-2, and TNF-α^9^. This further complicates the association between statins and PH. Additionally, there is currently a lack of evidence to confirm whether lipid-lowering drugs reduce portal pressure by alleviating intrahepatic vascular resistance, as well as liver fibrosis through anticoagulation.

Given the complex relationship between lipid-lowering drugs, coagulation function, and PH, observational studies were often obtained the contradictory results, which might cause by confounding factors and reverse causation. Furthermore, current researches have limited sample sizes and yield contradictory conclusions^9,10^. Therefore, this study adopted a new method, Mendelian randomization (MR), to analyze the complex relationship among these three factors. MR employed single nucleotide polymorphisms (SNPs) as instrumental variables (IVs) to infer causal associations^11^. It relies on following assumptions: (1) the IVs should be strong association with the exposure; (2) the IVs is related only to the exposure without other factors; and (3) the exposure affects the outcome solely through the IVs and not other pathways^11^. Since alleles are randomly inherited from parents and independent in transmission, MR can effectively eliminate environmental and other confounding factors, providing robust causal estimates^12^.

Based on this knowledge, we hypothesized that Low-Density Lipoprotein Cholesterol (LDL-c) reduction drugs reduce portal pressure through anticoagulant effects and employed MR to investigate the causal relationship between LDL-c reduction drugs and PH, using mediation analysis to explore the mediating role of coagulation factors. Ensuring the robustness of the results, meta-analysis was conducted to synthesize the conclusions from three datasets. Additionally, Phenome-wide MR (PWMR) was used to explore potential side effects and potential influence to liver function when treating PH with drug targets.

## Method

### Study design

**Fig. 1:**
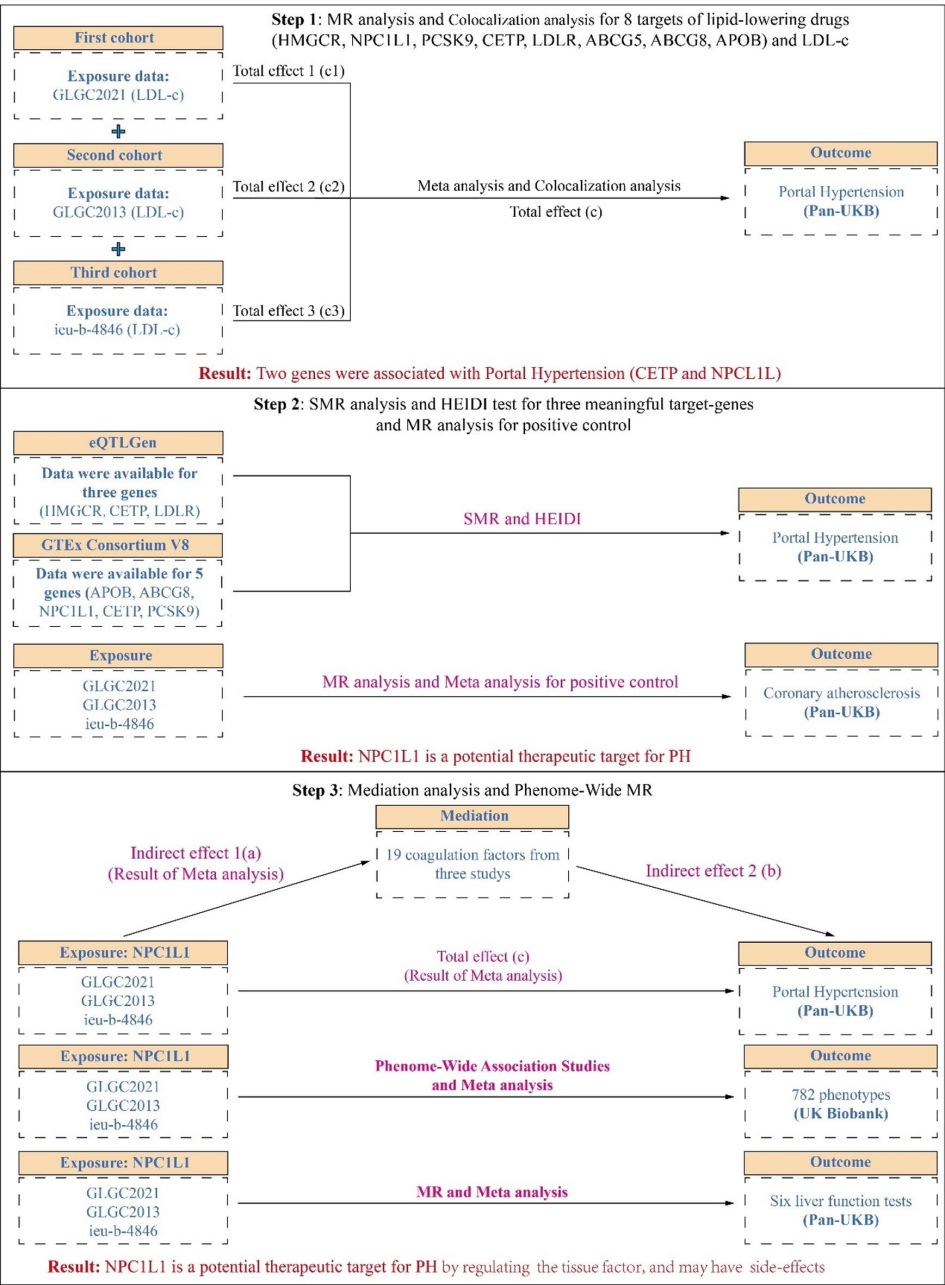
The flow chat of this study. In the first step, this study extracted eight lipid-lowering drug targets (ABCG5, ABCG8, APOB, HMGCR, LDLR, NPC1L1, CETP, and PCSK9) from three LDL-c studies, along with instrumental variables (IVs) for LDL-c after excluding the 8 lipid-lowering target genes. These were then analyzed through MR to obtain the overall effects on PH across each dataset (Total c1, c2, c3). A meta-analysis was conducted to combine the overall effects from the three datasets to derive a pooled overall effect (Total c). Additionally, colocalization analysis was performed on positive results to determine whether these genes share genetic variations with PH. The MR analysis results suggested a potential association between CETP and NPC1L1 with PH from genetic correlation perspective. In the second step, we extracted eight lipid-lowering drug genes from eQTLGen and GTEx V8 for SMR analysis and HEIDI test. From eQTLGen, we identified only HMGCR, CETP, and LDLR, while from GTEx V8, we extracted APOB, ABCG8, NPC1L1, CETP, and PCSK9. Furthermore, the IVs extracted from the three datasets were subjected to MR analysis concerning coronary atherosclerosis (CAD) to clarify whether these IVs accurately represent the LDL-c-lowering effects of the targets (positive controls). A meta-analysis was performed on the positive control results from these three datasets, identifying NPC1L1 as a potential therapeutic target for PH. In the third step, we conducted mediation analysis using NPC1L1 data from the three datasets along with data from 19 coagulation factors (NPC1L1 to coagulation factors: indirect effect a; coagulation factors to PH: indirect effect b). The results indicated that tissue factor may play a potential mediating role in the treatment of PH by NPC1L1. Additionally, we performed a PWMR analysis on the NPC1L1 gene to explore potential side effects of NPC1L1 inhibitors in the treatment of PH. In addition, the association between NPC1L1 and six liver function tests was also examined. LDL-c, low-density lipoprotein cholesterol; SMR, Summary-data-based Mendelian Randomization; HEIDI, heterogeneity in dependent instruments; GLGC, Global Lipids Genetics Consortium. PWMR, Phenome-wide Mendelian Randomization.

This study was performed following the STROBE-MR checklist to guide the implementation of the research (Table S1)^13^. The flowchart of research could be seen in Fig. 1.

### Lowering LDL-C targets

Consider that the guidelines recommend the use of statins, which are the classic LDL-c-lowering drugs. Therefore, we searched the previous literature for LDL-cholesterol-lowering drugs^14,15^. Finally, 8 LDL-c reduction drug targets were identified(3-hydroxy-3-methylglutaryl coenzyme A reductase [HMGCR], Niemann–Pick C1-Like 1 [NPC1L1], proprotein convertase subtilisin/kexin type 9 [PCSK9], cholesteryl ester transfer protein [CETP], Apolipoprotein B [APOB], ATP binding cassette subfamily G member 5 [ABCG5], ATP binding cassette subfamily G member 8 [ABCG8], low density lipoprotein cholesterol receptor [LDLR])^16,17^. The corresponding drug and the chromosomal location corresponding to the target gene can be found in Table S2. All chromosome and position mentioned above are based on Build GRCh37/hg 19, and all information were acquired from https://www.ncbi.nlm.nih.gov/gene.

### Data source

The dataset of PH were obtained from the Pan-UK Biobank, specifically selecting data from the European population^18^, which was sourced from the UK Biobank, aiming to include approximately 500,000 individuals aged 40 to 69. The dataset of PH included 725 cases and 408,402 healthy controls. For positive control analysis, the GWAS data of coronary atherosclerosis (CAD) also sourced from Pan-UK Biobank. It contained 23,888 cases of European descent and 382,052 controls. In addition, our study incorporated six liver function tests (LFTs) from European populations in the Pan-UKB dataset, including alkaline phosphatase (ALP), alanine aminotransferase (ALT), gamma-glutamyltransferase (GGT), aspartate aminotransferase (AST), direct bilirubin (DBIL), and total bilirubin (TBIL). Details of LFTs are provided in Table 1.

**Table 1:** Characteristics of GWAS data in this study.

Trait	Source	GWAS ID	PMID	Population	Sample size
**Exposure**					
low-density lipoprotein cholesterol	2021 Global Lipids Genetics Consortium (LDL-c)	–	34,887,591	European	1,320,016
low-density lipoprotein cholesterol	2013 Global Lipids Genetics Consortium (LDL-c)	–	24,097,068	European	173,082
low-density lipoprotein cholesterol	Within family GWAS consortium	ieu-b-4846	–	European	70,814
**Mediation**					
Coagulation factor Xa	IEU Open GWAS project	prot-a-1005	29,875,488	European	3,301
Coagulation Factor X	IEU Open GWAS project	prot-a-1006	29,875,488	European	3,301
Coagulation Factor VIII	IEU Open GWAS project	prot-a-1009	29,875,488	European	3,301
D-dimer	IEU Open GWAS project	prot-a-1086	29,875,488	European	3,301
Multiple coagulation factor deficiency protein 2	IEU Open GWAS project	prot-a-1867	29,875,488	European	3,301
Activated Protein C	IEU Open GWAS project	prot-a-2381	29,875,488	European	3,301
Antithrombin-III	IEU Open GWAS project	prot-a-2695	29,875,488	European	3,301
Plasminogen activator inhibitor 1	IEU Open GWAS project	prot-a-2696	29,875,488	European	3,301
coagulation factor III, tissue factor	IEU Open GWAS project	prot-b-10	28,369,058	European	3,301
coagulation factor II thrombin receptor	IEU Open GWAS project	prot-b-7	28,369,058	European	3,301
Coagulation Factor XI	IEU Open GWAS project	prot-c-2190_55_1	28,240,269	European	3,301
vWF	IEU Open GWAS project	prot-c-3050_7_2	28,240,269	European	3,301
Coagulation Factor Xa	IEU Open GWAS project	prot-c-3077_66_2	28,240,269	European	3,301
Coagulation Factor VII	IEU Open GWAS project	prot-c-3184_25_2	28,240,269	European	3,301
Coagulation Factor IX	IEU Open GWAS project	prot-c-4876_32_1	28,240,269	European	3,301
Coagulation Factor X	IEU Open GWAS project	prot-c-4878_3_1	28,240,269	European	3,301
Coagulation Factor V	IEU Open GWAS project	prot-c-4906_35_1	28,240,269	European	3,301
Coagulation Factor IXab	IEU Open GWAS project	prot-c-5307_12_3	28,240,269	European	3,301
**Outcome**					
Portal hypertension	Pan-UKB	–	–	European	409,127
**Positive control**					
Coronary atherosclerosis	Pan-UKB	–	–	European	405,940
**Liver function tests**					
Alkaline phosphatase	Pan-UKB	–	–	European	400,988
Alanine aminotransferase	Pan-UKB	–	–	European	400,822
Gamma glutamyltransferase	Pan-UKB	–	–	European	400,751
Aspartate aminotransferase	Pan-UKB	–	–	European	399,482
Direct bilirubin	Pan-UKB	–	–	European	340,934
Total bilirubin	Pan-UKB	–	–	European	399,286

2021 Global Lipids Genetics Consortium (GLGC2021), a large dataset of LDL-c, was acquired^19^, and as well as 2013 Global Lipids Genetics Consortium (GLGC2013)^20^, with sample sizes of 1,320,016 and 173,082, respectively. The dataset includes data from multiple ethnicities, however, we selected the European population data for analysis. To avoid sample overlap, we selected data that without UK Biobank dataset. Additionally, we searched in the IEU Open GWAS (Website: https://gwas.mrcieu.ac.uk/datasets/) and used another data as ieu-b-4846. This dataset includes 70,814 individuals of European ancestry.

The eQTLs of 8 targets for LDL-c reduction drugs in eQTLGen^21^ and Genotype-Tissue Expression (GTEx) Consortium V8 were selected, and Summary-data-based MR (SMR) format data of eQTL were obtained from a website (https://yanglab.westlake.edu.cn/software/smr/#DataResource). To elucidate the mediating effect of coagulation function between LDL-c reduction drugs and PH, we selected 19 coagulation-related proteins from three protein databases (Folkersen:2017^22^, Sun:2018^23^, Suhre:2017^24^). Detail ethnicity and number of individuals are provided in Table 1.

### Ethical approval

This study utilized publicly available data, with all ethical approvals accessible in the corresponding original articles.

### Instrumental variables

For the GAWS summary data of LDL-c, we used the following criteria to extract IVs: (1) SNPs within ± 250 kb of the genes were selected; (2) IVs were filtered, under a threshold of *P* < 5e-8, and performed clumping based on 1000G European data (phase 3) with an r^2^ threshold of < 0.3^25,26^. For the coagulation-related data, a threshold of *P* < 5e-6, r^2^ < 0.001, and 10,000 kb were used to extract IVs. Additionally, to exclude the direct effect of LDL-c on PH, IVs were selected using the following criteria: *P* < 5e-8, r² < 0.001, and a distance threshold of 10,000 kb. Subsequently, following the method described by Zhao et al., overlapping regions with eight target genes were removed, and the remaining IVs were used as LDL-c-specific IVs^27^. Following the calculation formula provided by Zhao et al., the individual SNPs with an F statistic < 10 were excluded, to avoid weak instrument bias, which might affect the results of the MR analysis^27^. Then, the all IVs were excluded which minor allele frequencies (MAF) is less than 0.01^28^. When harmonized IVs and outcome’s data, we did not use proxy SNPs and removed palindromic sequences.

### MR analysis and colocalization analysis

Inverse Variance Weighted (IVW), a algorithm of the “*TwoSampleMR*” (v0.5.8) package, was primarily used to estimate the association, and it is currently the most commonly used MR method^29^. In addition, Bayesian weighted MR (BWMR)^30^, and constrained maximum likelihood and model averaging-based MR (cML-MA)^31^ were used as supplementary analyses (*BWMR*: v0.1.1; and *cMR-ML*: v0.0.0.9). Considering that LDL-c reduction drugs inhibit the expression of the genes, we used the negative of the MR estimates to calculate the odds ratio (OR) and 95% confidence intervals (95%CI)^27^. This approach allows for a straightforward interpretation of the impact of LDL-c reduction drugs on PH. The following criteria were set to determine positive results: (1) consistent direction of estimates across the three methods (all estimates are either greater than 0 or less than 0)^32^; (2) correction of results using the Bonferroni correction procedure to mitigate potential false-positive results due to multiple testing^28^; A nominally significant threshold (0.05/N < *P* < 0.05, where N is the number of multiple tests) was considered suggestive evidence, while *P* < 0.05/N was considered significant association. Subsequently, the MR results of three data were combined by meta-analysis.

To identify association between LDL-c reduction drug targets and PH is due to shared genetic variation, we used colocalization analysis, based on the “*coloc*” package (version 5.2.2), to exclude the influence of linkage disequilibrium (LD)^33^. It contained five hypotheses: Firstly, there are no causal variants for either LDL-c reduction drug targets or PH within the genetic region (H0); Secondly, causal variants exist only for LDL-c reduction drug targets (H1); Thirdly, causal variants exist only for PH (H2); Fourthly, distinct causal variants exist for both the drug targets and PH (H3); Fifthly, LDL-c reduction drug targets and PH share causal variants (H4). It was considered that PH and targets share the same causal variants, when the posterior probability H4 (PPH4) > 75%^34^. We first utilized SNPs within 100 kb of the target and conducted the analysis using default parameters. Considering that colocalization analysis is sensitive to window size, which may impact the results, we employed a large window size (± 250kb)^35^.

### Supplementary analysis

Using IVs of 8 target genes in supplementary analysis, if the MR estimate indicates that inhibition of the target gene reduces the risk of CAD, it can be considered that the IVs effectively represents the target gene. If the association with CAD is not significant, it would indicate that the selected IVs cannot adequately represent the corresponding target genes. Through this approach, we were able to ascertain whether the selected IVs could represent the biological effects of the corresponding target genes. This further strengthens the validity of the aforementioned MR and colocalization analyses^27^. This is referred to as a positive control analysis.

In addition, to exclude the possibility that LDL-c reduction drugs treat PH by reducing LDL-c (Target genes - LDL-c - PH pathway), this study also selected IVs for LDL-c. After excluding the IVs associated with eight lipid-lowering drug targets form LDL-c, we conducted MR analysis between LDL-c and PH. If the MR analysis demonstrated an association between LDL-c and PH, it would suggest that the Target genes - LDL-c - PH pathway is valid; conversely, if no association is found, it would rule out LDL-c as a potential mediator.

### SMR analysis

SMR and heterogeneity in dependent instruments (HEIDI) test were used to further validate the association between targets and PH, which performed by SMR software (v1.3.1) with default parameters (*P* < 5e-8, MAF > 0.01)^36^, since previous study had shown that the SMR could achieve higher statistical power in large sample sizes, compared to conventional MR analysis^36^. The HEIDI test was employed to assess potential effects of LD on SMR results. The *P*_*HEIDI*_ is greater than 0.05 indicated that the association between PH and targets was not driven by LD^35^.

### Mediation analysis

Two-step MR was employed to detect the causal associations between meaningful LDL-c reduction drugs targets and coagulation factors, as well as the causal associations between intermediate factors and PH. The overall effect (c) could be decomposed into a direct effect (β) and an indirect effect (c’). The effect between the exposure and the intermediate factor is denoted as ‘a’, while the effect between the intermediate factor and the outcome is denoted as ‘b’. Thus, c’ = a * b. The direct effect (β) is then obtained as c - c’. As multiple databases were utilized in this study, a and c will have multiple values (GLGC2021: a1, c1; GLGC2013: a2, c2; and ieu-b-4846: a3, c3). To obtained the robust results, a meta-analysis was conducted to obtain the overall effect of ‘a’ and ‘c’. The meta-analysis-derived estimates were used in the mediation analysis to calculate the mediation effects and corresponding p-values.

### Proteome‑wide MR analysis

We conducted PWMR to further clarify the side-effects of LDL-c reduction drug targets on PH. The datasets were sourced from the UK Biobank (https://www.leelabsg.org/resources29)^37^. To maintain sufficient statistical power, we excluded phenotypes or symptoms with fewer than 500 cases, as well as PH, resulting in a final dataset of 782 phenotypes for PWMR (Table S3)^37^. We corrected the *P* value of results, to mitigate the risk of false positives, which relied on a significance threshold of *P* < 6.394e-05 (0.05/782). In addition, our study, considering the impact of lipid-lowering drugs on liver function, also conducted an association analysis between the therapeutic targets and six LFTs to explore the potential effects of potential drug-target genes on liver function.

### Sensitive analysis

We utilized Mendelian Randomization Pleiotropy RESidual Sum and Outlier (MR-PROSSO)^38^ and the MR-Egger intercept test^39^ to evaluate horizontal pleiotropy. MR-PROSSO was applied only when the number of SNPs exceeded 3 due to algorithm constraints^38^. Thus, when the number of IVs was 3 or fewer, the MR-Egger intercept test was primarily assessed horizontal pleiotropy. When the MR-PRESSO results indicated the presence of outliers, we used the IVW method from the “*RadialMR*” (v1.0) to identify and remove these outliers, followed by another round of MR analysis. Cochran’s Q test was implemented to detect heterogeneity^29^. Additionally, the statistical power of the MR analysis was calculated, following previous literature, by the website (https://sb452.shinyapps.io/power/)^40^. All analyses, in this study, were conducted using R software (version 4.2.3, China).

## Results

### Genetic instrument selection

The number and details of IVs were provided in Table S4-S6. For GLGC2021, we got 40, 21, 76, 294, 14, 88, 83, 57 and 56 SNPs within the HMGCR, NPC1L1, PCSK9, LDL-c, CETP, LDLR, APOB, ABCG5 and ABCG8 (Table S4). For GLGC2013, we obtained 162 SNPs from GWAS summary data, including 8 SNPs for HMGCR, 3 SNPs for NPC1L1, 16 SNPs for PCSK9, 69 SNPs for LDL-c, 4 SNPs for CETP, 30 SNPs for APOB, 7 SNPs for ABCG5, 7 SNPs for ABCG8 and 18 SNPs for LDLR (Table S5). For ieu-b-4846, we acquired 126 SNPs from this exposure GWAS data, including 6 SNPs for HMGCR, 3 SNPs for NPC1L1, 13 SNPs for PCSK9, 42 SNPs for LDL-c, 3 SNPs for CETP, 24 SNPs for APOB, 8 SNPs for ABCG5, 7 SNPs for ABCG8 and 21 SNPs for LDLR (Table S6). In addition, we also obtained 110 SNPs of coagulation factors after removing the IVs of NPC1L1 and detail information could find in Table S7.

### MR analysis and colocalization analysis

**Fig. 2:**
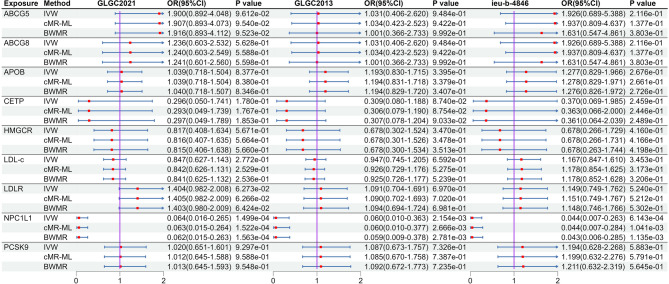
The result of MR analysis between lipid-lowering drugs and portal hypertension. Instrumental variables related to target genes and LDL-c were extracted from three large LDL-c databases (GLGC2021, GLGC2013, ieu-b-4846). Subsequently, the association between these targets and portal hypertension was analyzed.

**Fig. 3:**
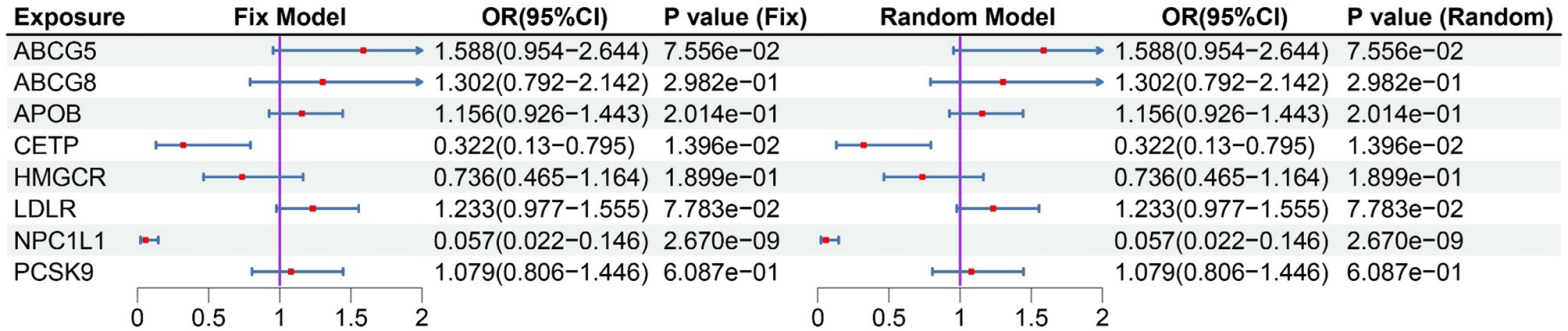
Meta-analysis results between lipid-lowering drug targets and portal hypertension. The left side is the fixed effect model, and the right side is the random effect model. Meta-analysis results showed that there is a significant causal association between NPC1L1 and portal hypertension (*P* < 0.05/9), while there is a suggestive causal association between CETP and portal hypertension. Other target genes showed no correlation (*P* > 0.05).

After MR analysis (Table S8), we found that NPC1L1 was significant associated with PH (OR: 0.064, 95%CI: 0.016–0.265, *P*_*IVW*_ = 1.499e-4) in the GLGC2021, and this result was repeated in the GLGC2013 (OR: 0.060, 95%CI: 0.010–0.363, *P*_*IVW*_ = 2.153e-3) and ieu-b-4846 (OR: 0.044, 95%CI: 0.007–0.263, *P*_*IVW*_ = 6.143e-4). All these results were significant association (*P* < 5.556e-03[0.05/9]). The other target-genes did not show significant association across all three datasets (Fig. 2). Sensitivity analysis indicated no heterogeneity (*P*_Cochran’s Q test_ > 0.05) and pleiotropy (*P*_*MR−PRESSO global test*_ and *P*_*MR Egger intercept test*_ > 0.05) in the MR analysis results (Table S9). The estimates from the three methods remained consistent (Fig. 2). After meta-analysis, we found that two targets (CETP and NPCL1L) were associated with PH, and only NPCL1L was significant association (OR: 0.057, 95%CI: 0.022–0.146, *P*_*Meta of IVW*_ = 2.670e-09), CETP was suggestive associated (*P*_*Meta of IVW*_ = 0.014, Fig. 3). The statistical power of the MR analysis is shown in the Table S10.

**Fig. 4:**
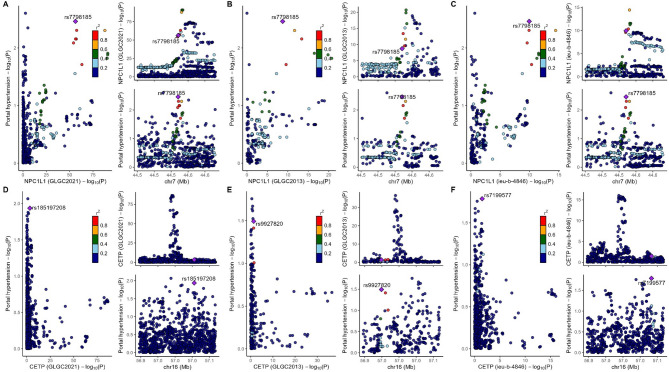
The result of colocalization analysis (100 kb). SNPs within 100 kb of the target genes (CETP and NPC1L1) were utilized and conducted the analysis using default parameters. Three LDL-c databases (2021 Global Lipids Genetics Consortium [2021GLGC], 2013 Global Lipids Genetics Consortium [2013GLGC], ieu-b-4846) were used for colocalization analysis with portal hypertension (PH). (A) 2021GLGC: NPC1L1 and PH; (B) 2013GLGC: NPC1L1 and PH; (C) ieu-b-4846: NPC1L1 and PH; (D) 2021GLGC: CETP and PH; (E) 2013GLGC: CETP and PH; (F) ieu-b-4846: CETP and PH.

**Fig. 5:**
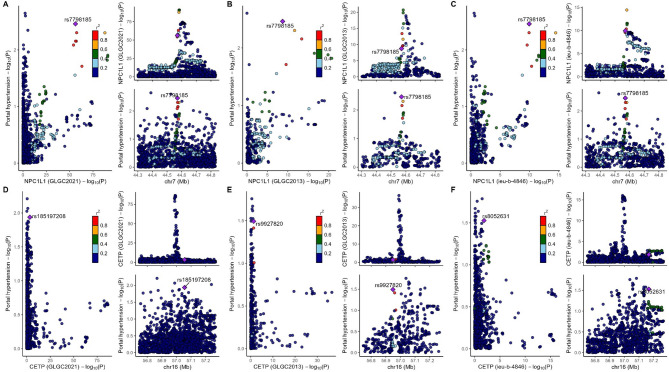
The result of colocalization analysis (250 kb). SNPs within 250 kb of the target genes (CETP and NPC1L1) were utilized and conducted the analysis using default parameters. Three LDL-c databases (2021 Global Lipids Genetics Consortium [2021GLGC], 2013 Global Lipids Genetics Consortium [2013GLGC], ieu-b-4846) were used for colocalization analysis with portal hypertension (PH). (A) 2021GLGC: NPC1L1 and PH; (B) 2013GLGC: NPC1L1 and PH; (C) ieu-b-4846: NPC1L1 and PH; (D) 2021GLGC: CETP and PH; (E) 2013GLGC: CETP and PH; (F) ieu-b-4846: CETP and PH.

Subsequently, we performed colocalization analysis on two genes (window size = 100 kb, Fig. 4, Table S11), which revealed that no target-genes share genetic variants with PH (all PPH4 < 75%). Then, we used the larger window size (250 kb) to replicate the colocalization analysis, and the results displayed the same with before (Table S11, Fig. 5).

### Supplementary analysis

**Fig. 6:**
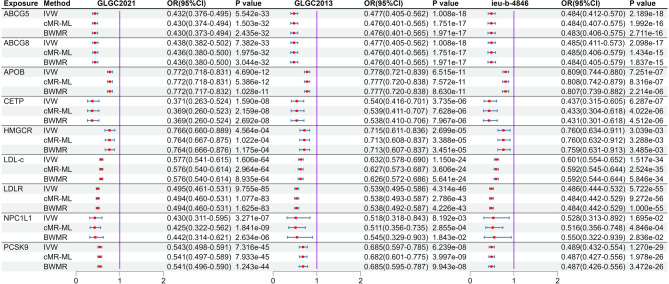
The result of MR analysis of positive analysis. Using coronary atherosclerosis (CAD) as a positive control, all lipid-lowering drug targets were shown to reduce the risk of coronary atherosclerosis (CAD). This indicates that the instrumental variables are capable of representing the biological effects of the corresponding target genes.

**Fig. 7:**
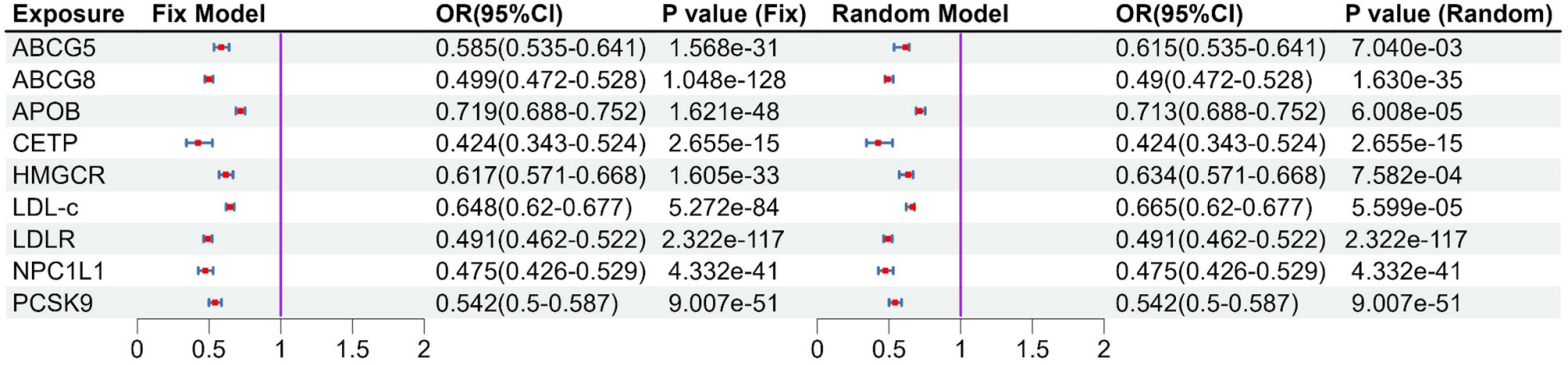
The result of meta-analysis of positive analysis. The left side is the fixed effect model, and the right side is the random effect model. Meta-analysis results showed that all targets were significant causal association with CA (*P* < 0.05/9).

Using CAD as a positive control outcome, we found that inhibiting the 8 LDL-c reduction drug targets were linked to a decreased risk of CAD. (Fig. 6). This result demonstrated a suppressive effect across GLGC2021, GLGC2013, and ieu-b-4846 (Fig. 6). Subsequently, a meta-analysis was employed on the results for NPC1L1. The findings indicated that NPC1L1 inhibitors have a protective effect against CAD (Fix model: OR: 0.475, 95% CI: 0.425–0.530, Fig. 7). This result demonstrates that IVs could substitute for NPC1L1. Subsequent analyses revealed that there was no genetic-level correlation between LDL-c and PH in any of the three datasets. The results of the meta-analysis also indicated the same conclusion (Fig. 2, and Fig. 3). This suggests that the potential therapeutic effect of NPC1L1 on PH is not mediated through the reduction of LDL-c.

### SMR analysis

We performed the SMR analysis and HEIDI test to verify the results of traditional MR analysis. SMR analysis and HEIDI test demonstrated that only NPC1L1 showed a significant association (OR: 0.648, 95%CI: 0.472–0.891, *P*_*SMR*_ = 7.502e-3, *P*_*HEIDI*_ = 0.747, Table S12). Considering the large sample size, the SMR method can achieve higher statistical power compared to traditional methods. Therefore, we identified NPC1L1 as a meaningful therapeutic target for PH.

### Mediation analysis

**Fig. 8:**
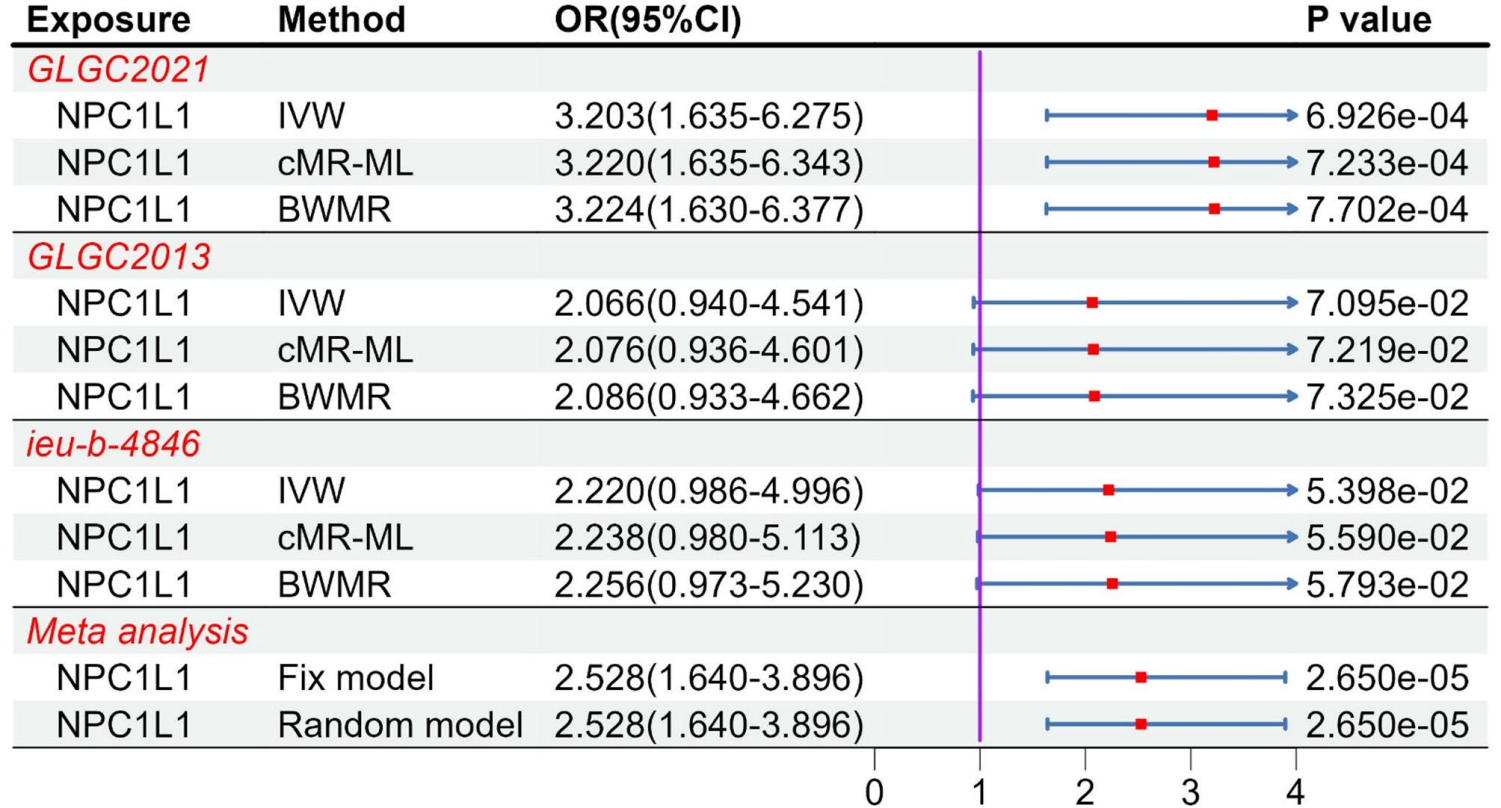
The result of MR analysis between NPC1L1 and tissue factor. MR analysis revealed that only GLGC2021 showed an association between NPC1L1 and tissue factor, while the GLGC2013 and ieu-b-4846 did not demonstrate this relationship. A subsequent meta-analysis of the three databases confirmed the association between NPC1L1 and tissue factor.

By using of two-step MR to examine the relationship between the target gene and coagulation function, NPC1L1 showed genetically association with tissue factor (OR: 3.203, 95%CI: 1.635–6.275, *P*_*IVW*_ = 6.926e-4 [less than 0.05/18]) only in the GLGC2021 (Table S13-S14, Fig. 8). The GLGC2013 and ieu-b-4846 did not demonstrate genetically association between NPC1L1 and tissue factor (*P*_*IVW*_ > 0.05). Furthermore, we didn’t find other factors which were genetically associated with NPC1L1. Subsequently, results of meta-analysis from three databases indicated a significant association between NPC1L1 and tissue factor (Fix model: OR: 2.528, 95%CI: 1.640–3.896, Fig. 8). We considered the meta-analysis estimate between exposure and outcome as the total effect (c). The meta-analyzed estimate between NPC1L1 and mediator was defined as indirect effect A (a), and the estimate between mediator and outcome as indirect effect B (b). The final mediation effect results were displayed in the Table 2. The *P*-value of mediation analysis indicated significant mediation effect (*P*_*Mediation*_ = 0.01). In the two-step analysis, the estimates from the three methods were consistent, with no evidence of heterogeneity or horizontal pleiotropy (Table S9). The statistical power was shown in the Table S10.

**Table 2:** The results of mediation analysis.

Exposure	Mediation	Outcome	Total effect (c, 95%CI)	Indirect effect (a, 95%)	Indirect effect (b, 95%CI)	Mediation (c’, 95%CI)	Proportion	*P* value
NPC1L1	coagulation factor III, tissue factor	Portal hypertension	−2.867(−3.812 to −1.923)	−0.922(−1.370 to −0.474)	0.922(0.475 to 1.369)	−0.576(−0.906 to −0.246)	18.52%	0.01

### Proteome‑wide MR analysis

**Fig. 9:**
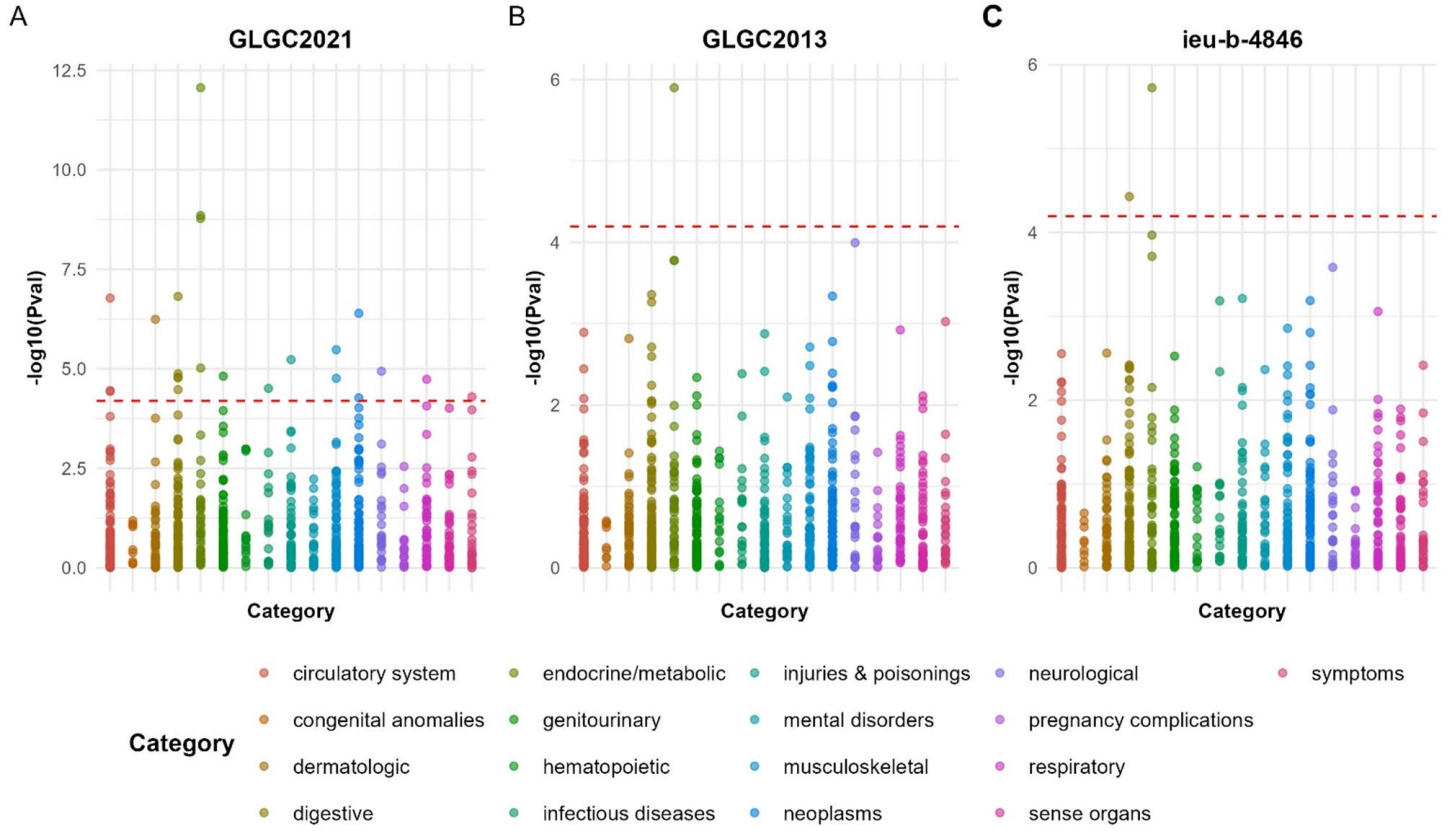
The results of Phenome-Wide MR analysis. The PWMR analysis indicated that, at the genetic level, 23 (GLGC2021), 1 (GLGC2013), and 2 (ieu-b-4846) diseases or symptoms were significantly associated with NPC1L1, respectively. In the GLGC2021, NPC1L1 inhibitors may reduce the risk of the following diseases or symptoms, including: respiratory abnormalities, pancreatic cancer, obstruction of the bile duct, adverse effects caused by opiates and related narcotics during therapeutic use, malposition and malpresentation of the fetus or obstruction, Bladder neck obstruction, hypovolemia, unstable angina (intermediate coronary syndrome), acquired toe deformities, ulcer of the esophagus, coronary atherosclerosis, hypercholesterolemia, disorders of lipoid metabolism, hyperlipidemia, ischemic heart disease. In addition, it may also increase the risk of the diseases or conditions, including gastritis and duodenitis, viral infection, sciatica, migraine, actinic keratosis, other acquired musculoskeletal deformities, abnormal findings on examination of the gastrointestinal tract or abdominal area, cancer of the esophagus. In both GLGC2013 and ieu-b-4846, the therapeutic effect of NPC1L1 inhibitors was observed solely for hyperlipidemia (*P* < 0.05/782).

Among the 782 phenotypes analyzed, 23 (GLGC2021), 1 (GLGC2013), and 2 (ieu-b-4846) diseases or symptoms were significantly associated with NPC1L1, respectively (Table S15, Fig. 9). In the GLGC2021, NPC1L1 inhibitors may reduce the risk of the following diseases or symptoms, including: respiratory abnormalities, pancreatic cancer, obstruction of the bile duct, adverse effects caused by opiates and related narcotics during therapeutic use, malposition and malpresentation of the fetus or obstruction, Bladder neck obstruction, hypovolemia, unstable angina (intermediate coronary syndrome), acquired toe deformities, ulcer of the esophagus, coronary atherosclerosis, hypercholesterolemia, disorders of lipoid metabolism, hyperlipidemia, ischemic heart disease. In addition, it may also increase the risk of the diseases or conditions, including gastritis and duodenitis, viral infection, sciatica, migraine, actinic keratosis, other acquired musculoskeletal deformities, abnormal findings on examination of the gastrointestinal tract or abdominal area, cancer of the esophagus. In both the GLGC2013 and ieu-b-4846, the therapeutic effect of NPC1L1 inhibitors was observed solely for hyperlipidemia (*P* < 0.05/782). Sensitivity analysis indicated no exist heterogeneity or horizontal pleiotropy in significant disease or symptoms (Table S16). Subsequently, we conducted a meta-analysis on 23 potential complications identified in the GLGC2021. After screening using the threshold of 2.131e-05 (0.05/782/3), 23 phenotypes remained associated with NPC1L1 (Table S17). In all three datasets, NPC1L1 inhibitors consistently demonstrated an inhibitory effect on hyperlipidemia.

**Fig. 10:**
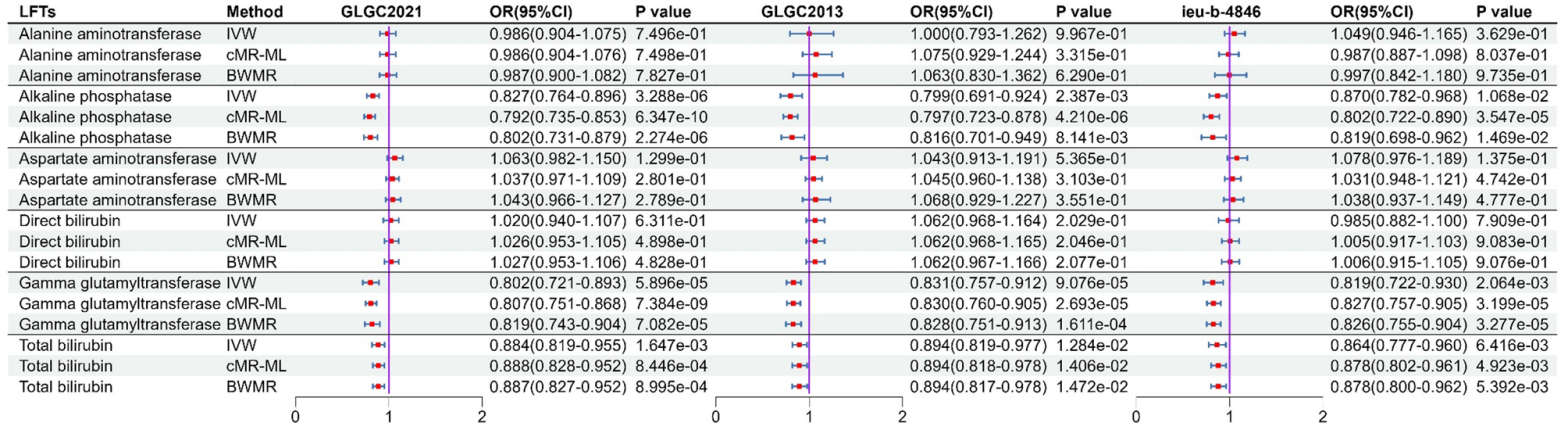
The result of MR analysis between NPC1L1 and LFTs. MR analysis revealed association between NPC1L1 and ALP, GGT, TBIL. However, the MR results for GGT exhibited heterogeneity and horizontal pleiotropy, rendering them unreliable. Ultimately, genetic prediction confirmed that NPC1L1 inhibitors have a protective effect on ALP and TBIL.

**Fig. 11:**
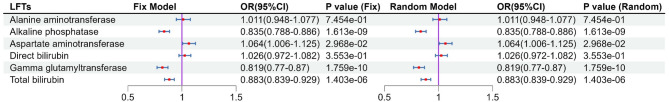
Meta-analysis results between NPC1L1 and liver function tests. Meta analysis revealed association between NPC1L1 and ALP, GGT, TBIL. However, the MR results for GGT exhibited heterogeneity and horizontal pleiotropy, rendering them unreliable. Ultimately, genetic prediction confirmed that NPC1L1 inhibitors have a protective effect on ALP and TBIL.

The MR analysis between NPC1L1 and LFTs genetically predicted that NPC1L1 inhibitors would reduce ALP, GGT, and TBIL (Fig. 10). However, only GGT showed significant associations across all three datasets (*P* < 0.05/6). In contrast, no genetically predicted associations were observed between NPC1L1 and ALT, AST, or DBIL (Fig. 10, Table S18). Furthermore, meta-analysis results further confirmed that ALP, GGT, and TBIL were genetically associated with NPC1L1 (Fig. 11, Table S19). Due to the presence of heterogeneity and horizontal pleiotropy in the sensitivity analysis for GGT, the results are deemed unreliable (Table S20). Consequently, this study only concludes that ALP and TBIL exhibit genetic associations with NPC1L1.

## Discussion

To the best of our knowledge, this study is the first systematic exploration of the causal relationship between LDL-c reduction drugs and PH from a genetic perspective. The conclusions drawn from the aggregation of GWAS and eQTLs data provide potential evidence for causal inference between LDL-c reduction drugs and PH. By using genetic variation as IVs to infer causality, our study eliminates the influence of confounding factors (such as the severity of cirrhosis, liver function grading, and etc.) and the reverse causality that conventional observational studies cannot exclude. Our study employed three different LDL-c databases and conducted a meta-analysis. Therefore, the conclusions derived from our study are relatively reliable, demonstrating a potential causal link from a genetic standpoint.

The causes of intrahepatic micro-thrombosis are complex, potentially involving inflammation, hepatic stellate cells, and multiple other factors. The outcome is increased intrahepatic vascular resistance and elevated portal pressure^3^. Animal study had shown that oral administration of rivaroxaban in cirrhotic mice reduces portal pressure, which is related to decreased intrahepatic vascular resistance and reduced intrahepatic micro-thrombosis^41^. Another animal study using low-molecular-weight heparin demonstrated that both short-term (1 week) and long-term (3 weeks) use of anticoagulants effectively relieved portal pressure and intrahepatic vascular resistance without affecting mean arterial pressure^4^. Notably, a study on the simvastatin also showed that both short-term and long-term use could improve portal pressure^42^. However, our study did not find that statins’ target (HMGCR) has a therapeutic effect on PH. This does not mean that statins have no therapeutic effect on PH, but only that they do not act through conventional statin targets (HMGCR).

Our study indicated that NPC1L1 inhibitors are potential therapeutic drugs for PH. The primary function of NPC1L1 is to participate in the transport and absorption of cholesterol in the intestine and liver, as well as the absorption of vitamin K. As early as 2015, Takada et al. found that NPC1L1 is a key protein for intestinal vitamin K absorption. Compared to using warfarin alone, the combination of NPC1L1 inhibitors with warfarin can significantly reduce intrahepatic vitamin K levels. Vitamin K is a micronutrient that promotes coagulation, and its deficiency can lead to coagulation disorders. Vitamin K mainly participates in the extrinsic coagulation pathway, involving tissue factors. Clinical studies also displayed the relationship between coagulation-related indicators and HVPG. For example, a study, in 2022, showed that the ratio of procoagulant factor VIII to anticoagulant protein C is significantly associated with HVPG in cirrhotic patients^43^. Clinical guidelines also recommend anticoagulation therapy for patients with Child-Pugh A or B without clear indications of bleeding, as this can improve prognosis and long-term survival rates^2^.

NPC1L1 inhibitors may also prevent the occurrence of portal vein thrombosis (PVT), a severe complication of PH. PVT can reduce overall survival in patients with cirrhosis^44^. However, the mechanisms behind PVT formation remain controversial^44^. During PH, the portal vein is in a fragile balance, with decreased platelets and reduced coagulation and anticoagulation factors^44^. A prospective study indicated that reduced portal blood flow velocity, thrombocytopenia, and acute venous bleeding are risk factors for PVT^45^. Our result suggested that NPC1L1 inhibitors may serve as potential preventive agents for PVT, as they not only lower portal pressure but also modulate coagulation functions to further prevent PVT. However, further studies are needed to confirm these findings.

Although our study, from the perspective of genetic prediction, reveals the therapeutic effects of NPC1L1 inhibitors on PH, there are still some limitations. (1) The databases we used were all from European populations, so whether the conclusions can be extended to other ethnic groups requires further research; (2) We used a relatively lenient *P*-value cutoff (5e-6) as a criterion for selecting coagulation factor instrumental variables, which may influence the conclusions; (3) We used the B-H method to correct results, which, while reducing false positives, may have hindered the discovery of potential associations, leading to false negatives. (4) Some studies use the Phenoscanner V2 to check if the IVs are associated with outcomes through other ways^46^. However, the website is currently unavailable. Therefore, we used RadialMR to remove outliers and employed MR-PRESSO and the MR-Egger intercept test to assess potential horizontal pleiotropy. (5) Due to the lack of protein quantitative trait loci (pQTL) data and eQTL data specific to the digestive system, this study was unable to analyze the impact of intrahepatic target genes expression changes on PH or the alterations in downstream proteins associated with the gene. Further research is required to clarify the conclusions of our study.

## Conclusions

Using publicly available genetic data, this study is the first to establish an association between coagulation function, portal hypertension, and LDL-c reduction drugs from the perspective of genetic prediction. Our research highlights the potentially therapeutic effects of NPC1L1 inhibitors on portal hypertension, potentially through the inhibition of tissue factor.

## Electronic supplementary material

Below is the link to the electronic supplementary material.

Supplementary Material 1

## Data Availability

The GWAS data could find in IEU open GWAS project (https://gwas.mrcieu.ac.uk/datasets/), Global Lipids Genetics Consortium (https://csg.sph.umich.edu/willer/public) and Pan-UK Biobank (https://pan.ukbb.broadinstitute.org/downloads). SMR format data of eQTL were obtained from website (https://yanglab.westlake.edu.cn/software/smr/#DataResource).

## References

[CR1] 1. Gines, P. *et al.* Liver cirrhosis. *Lancet* **398**, 1359–1376, doi:10.1016/S0140-6736(21)01374-X (2021). 10.1016/S0140-6736(21)01374-X 34543610

[CR2] 2. de Franchis, R. *et al.* Baveno VII - Renewing consensus in portal hypertension. *J Hepatol* **76**, 959–974, doi:10.1016/j.jhep.2021.12.022 (2022). 10.1016/j.jhep.2021.12.022 PMC11090185 35120736

[CR3] 3. Airola, C. *et al.* Microvascular Thrombosis and Liver Fibrosis Progression: Mechanisms and Clinical Applications. *Cells* **12**, doi:10.3390/cells12131712 (2023). 10.3390/cells12131712 PMC10341358 37443746

[CR4] 4. Cerini, F. *et al.* Enoxaparin reduces hepatic vascular resistance and portal pressure in cirrhotic rats. *J Hepatol* **64**, 834–842, doi:10.1016/j.jhep.2015.12.003 (2016). 10.1016/j.jhep.2015.12.003 26686269

[CR5] 5. Bordbar, M. *et al.* Differential effect of statin use on coagulation markers: an active comparative analysis in the NEO study. *Thromb J* **19**, 45, doi:10.1186/s12959-021-00299-2 (2021). 10.1186/s12959-021-00299-2 PMC8237446 34176487

[CR6] 6. Takada, T. *et al.* NPC1L1 is a key regulator of intestinal vitamin K absorption and a modulator of warfarin therapy. *Sci Transl Med* **7**, 275ra223, doi:10.1126/scitranslmed.3010329 (2015). 10.1126/scitranslmed.3010329 25696002

[CR7] 7. Pollo-Flores, P. *et al.* Three months of simvastatin therapy vs. placebo for severe portal hypertension in cirrhosis: A randomized controlled trial. *Dig Liver Dis* **47**, 957–963, doi:10.1016/j.dld.2015.07.156 (2015). 10.1016/j.dld.2015.07.156 26321186

[CR8] 8. Bishnu, S. *et al.* Effects of atorvastatin on portal hemodynamics and clinical outcomes in patients with cirrhosis with portal hypertension: a proof-of-concept study. *Eur J Gastroenterol Hepatol* **30**, 54–59, doi:10.1097/MEG.0000000000001006 (2018). 10.1097/MEG.0000000000001006 29099421

[CR9] 9. Kronborg, T. M. *et al.* Atorvastatin for patients with cirrhosis. A randomized, placebo-controlled trial. *Hepatology Communications* **7**, doi:10.1097/hc9.0000000000000332 (2023). 10.1097/HC9.0000000000000332 PMC10697620 38051553

[CR10] 10. Sharpton, S. R. & Loomba, R. Emerging role of statin therapy in the prevention and management of cirrhosis, portal hypertension, and HCC. *Hepatology* **78**, 1896–1906, doi:10.1097/HEP.0000000000000278 (2023). 10.1097/HEP.0000000000000278 37013380

[CR11] 11. Kjaergaard, A. D., Smith, G. D. & Stewart, P. Mendelian Randomization Studies in Endocrinology: Raising the Quality Bar for Submissions and Publications in The Journal of Clinical Endocrinology & Metabolism. *J Clin Endocrinol Metab* **109**, 1–3, doi:10.1210/clinem/dgad569 (2023). 10.1210/clinem/dgad569 37796951

[CR12] 12. Davies, N. M., Holmes, M. V. & Davey Smith, G. Reading Mendelian randomisation studies: a guide, glossary, and checklist for clinicians. *BMJ* **362**, k601, doi:10.1136/bmj.k601 (2018). 10.1136/bmj.k601 PMC6041728 30002074

[CR13] 13. Skrivankova, V. W. *et al.* Strengthening the Reporting of Observational Studies in Epidemiology Using Mendelian Randomization: The STROBE-MR Statement. *JAMA* **326**, 1614–1621, doi:10.1001/jama.2021.18236 (2021). 10.1001/jama.2021.18236 34698778

[CR14] 14. Cai, G., Liu, J., Cai, M. & Shao, L. Exploring the causal effect between lipid-modifying drugs and idiopathic pulmonary fibrosis: a drug-target Mendelian randomization study. *Lipids in Health and Disease* **23**, doi:10.1186/s12944-024-02218-6 (2024). 10.1186/s12944-024-02218-6 PMC11293199 39090671

[CR15] 15. Ridker, P. M. LDL cholesterol: controversies and future therapeutic directions. *Lancet* **384**, 607–617, doi:10.1016/S0140-6736(14)61009-6 (2014). 10.1016/S0140-6736(14)61009-6 25131980

[CR16] 16. Li, Z. *et al.* Genetic association of lipids and lipid-lowering drug target genes with non-alcoholic fatty liver disease. *eBioMedicine* **90**, doi:10.1016/j.ebiom.2023.104543 (2023). 10.1016/j.ebiom.2023.104543 PMC10070091 37002989

[CR17] 17. Bi, Y., Zhu, Y., Tang, S. & Huang, Y. Lipids, lipid-modifying drug target genes and migraine: a Mendelian randomization study. *The Journal of Headache and Pain* **24**, doi:10.1186/s10194-023-01633-x (2023). 10.1186/s10194-023-01633-x PMC10439594 37596566

[CR18] 18. Karczewski, K. J. *et al.* Pan-UK Biobank GWAS improves discovery, analysis of genetic architecture, and resolution into ancestry-enriched effects. *medRxiv*, doi:10.1101/2024.03.13.24303864 (2024).

[CR19] 19. Graham, S. E. *et al.* The power of genetic diversity in genome-wide association studies of lipids. *Nature* **600**, 675–679, doi:10.1038/s41586-021-04064-3 (2021). 10.1038/s41586-021-04064-3 PMC8730582 34887591

[CR20] 20. Willer, C. J. *et al.* Discovery and refinement of loci associated with lipid levels. *Nat Genet* **45**, 1274–1283, doi:10.1038/ng.2797 (2013). 10.1038/ng.2797 PMC3838666 24097068

[CR21] 21. Vosa, U. *et al.* Large-scale cis- and trans-eQTL analyses identify thousands of genetic loci and polygenic scores that regulate blood gene expression. *Nat Genet* **53**, 1300–1310, doi:10.1038/s41588-021-00913-z (2021). 10.1038/s41588-021-00913-z PMC8432599 34475573

[CR22] 22. Folkersen, L. *et al.* Mapping of 79 loci for 83 plasma protein biomarkers in cardiovascular disease. *PLoS Genet* **13**, e1006706, doi:10.1371/journal.pgen.1006706 (2017). 10.1371/journal.pgen.1006706 PMC5393901 28369058

[CR23] 23. Suhre, K. *et al.* Connecting genetic risk to disease end points through the human blood plasma proteome. *Nat Commun* **8**, 14357, doi:10.1038/ncomms14357 (2017). 10.1038/ncomms14357 PMC5333359 28240269

[CR24] 24. Sun, B. B. *et al.* Genomic atlas of the human plasma proteome. *Nature* **558**, 73–79, doi:10.1038/s41586-018-0175-2 (2018). 10.1038/s41586-018-0175-2 PMC6697541 29875488

[CR25] 25. Fairley, S., Lowy-Gallego, E., Perry, E. & Flicek, P. The International Genome Sample Resource (IGSR) collection of open human genomic variation resources. *Nucleic Acids Res* **48**, D941-D947, doi:10.1093/nar/gkz836 (2020). 10.1093/nar/gkz836 PMC6943028 31584097

[CR26] 26. Gao, X. *et al.* Genetic association of lipid-lowering drugs with aortic aneurysms: a Mendelian randomization study. *Eur J Prev Cardiol* **31**, 1132–1140, doi:10.1093/eurjpc/zwae044 (2024). 10.1093/eurjpc/zwae044 38302118

[CR27] 27. Zhao, S. S., Yiu, Z. Z. N., Barton, A. & Bowes, J. Association of Lipid-Lowering Drugs With Risk of Psoriasis: A Mendelian Randomization Study. *JAMA Dermatol* **159**, 275–280, doi:10.1001/jamadermatol.2022.6051 (2023). 10.1001/jamadermatol.2022.6051 PMC9878432 36696131

[CR28] 28. Yu, K. *et al.* Assessment of bidirectional relationships between brain imaging-derived phenotypes and stroke: a Mendelian randomization study. *BMC Med* **21**, 271, doi:10.1186/s12916-023-02982-9 (2023). 10.1186/s12916-023-02982-9 PMC10369749 37491271

[CR29] 29. Burgess, S., Butterworth, A. & Thompson, S. G. Mendelian randomization analysis with multiple genetic variants using summarized data. *Genet Epidemiol* **37**, 658–665, doi:10.1002/gepi.21758 (2013). 10.1002/gepi.21758 PMC4377079 24114802

[CR30] 30. Zhao, J. *et al.* Bayesian weighted Mendelian randomization for causal inference based on summary statistics. *Bioinformatics* **36**, 1501–1508, doi:10.1093/bioinformatics/btz749 (2020). 10.1093/bioinformatics/btz749 31593215

[CR31] 31. Xue, H., Shen, X. & Pan, W. Constrained maximum likelihood-based Mendelian randomization robust to both correlated and uncorrelated pleiotropic effects. *Am J Hum Genet* **108**, 1251–1269, doi:10.1016/j.ajhg.2021.05.014 (2021). 10.1016/j.ajhg.2021.05.014 PMC8322939 34214446

[CR32] 32. Chen, X. *et al.* Depression and prostate cancer risk: A Mendelian randomization study. *Cancer Med* **9**, 9160–9167, doi:10.1002/cam4.3493 (2020). 10.1002/cam4.3493 PMC7724297 33027558

[CR33] 33. Williams, S. M. *et al.* Bayesian Test for Colocalisation between Pairs of Genetic Association Studies Using Summary Statistics. *PLoS Genetics* **10**, doi:10.1371/journal.pgen.1004383 (2014). 10.1371/journal.pgen.1004383 PMC4022491 24830394

[CR34] 34. Luo, S. *et al.* Effects of putative metformin targets on phenotypic age and leukocyte telomere length: a mendelian randomisation study using data from the UK Biobank. *Lancet Healthy Longev* **4**, e337-e344, doi:10.1016/S2666-7568(23)00085-5 (2023). 10.1016/S2666-7568(23)00085-5 37421961

[CR35] 35. Sun, J. *et al.* Identification of novel protein biomarkers and drug targets for colorectal cancer by integrating human plasma proteome with genome. *Genome Med* **15**, 75, doi:10.1186/s13073-023-01229-9 (2023). 10.1186/s13073-023-01229-9 PMC10508028 37726845

[CR36] 36. Zhu, Z. *et al.* Integration of summary data from GWAS and eQTL studies predicts complex trait gene targets. *Nat Genet* **48**, 481–487, doi:10.1038/ng.3538 (2016). 10.1038/ng.3538 27019110

[CR37] 37. Zhou, W. *et al.* Efficiently controlling for case-control imbalance and sample relatedness in large-scale genetic association studies. *Nat Genet* **50**, 1335–1341, doi:10.1038/s41588-018-0184-y (2018). 10.1038/s41588-018-0184-y PMC6119127 30104761

[CR38] 38. Verbanck, M., Chen, C. Y., Neale, B. & Do, R. Detection of widespread horizontal pleiotropy in causal relationships inferred from Mendelian randomization between complex traits and diseases. *Nat Genet* **50**, 693–698, doi:10.1038/s41588-018-0099-7 (2018). 10.1038/s41588-018-0099-7 PMC6083837 29686387

[CR39] 39. Bowden, J., Davey Smith, G. & Burgess, S. Mendelian randomization with invalid instruments: effect estimation and bias detection through Egger regression. *Int J Epidemiol* **44**, 512–525, doi:10.1093/ije/dyv080 (2015). 10.1093/ije/dyv080 PMC4469799 26050253

[CR40] 40. Pierce, B. L., Ahsan, H. & Vanderweele, T. J. Power and instrument strength requirements for Mendelian randomization studies using multiple genetic variants. *Int J Epidemiol* **40**, 740–752, doi:10.1093/ije/dyq151 (2011). 10.1093/ije/dyq151 PMC3147064 20813862

[CR41] 41. Vilaseca, M. *et al.* The anticoagulant rivaroxaban lowers portal hypertension in cirrhotic rats mainly by deactivating hepatic stellate cells. *Hepatology* **65**, 2031–2044, doi:10.1002/hep.29084 (2017). 10.1002/hep.29084 28142199

[CR42] 42. Abraldes, J. G. *et al.* Simvastatin lowers portal pressure in patients with cirrhosis and portal hypertension: a randomized controlled trial. *Gastroenterology* **136**, 1651–1658, doi:10.1053/j.gastro.2009.01.043 (2009). 10.1053/j.gastro.2009.01.043 19208350

[CR43] 43. Scheiner, B. *et al.* Factor VIII/protein C ratio independently predicts liver-related events but does not indicate a hypercoagulable state in ACLD. *J Hepatol* **76**, 1090–1099, doi:10.1016/j.jhep.2021.12.038 (2022). 10.1016/j.jhep.2021.12.038 35066090

[CR44] 44. Elkrief, L. *et al.* Portal vein thrombosis: diagnosis, management, and endpoints for future clinical studies. *Lancet Gastroenterol Hepatol* **9**, 859–883, doi:10.1016/S2468-1253(24)00155-9 (2024). 10.1016/S2468-1253(24)00155-9 38996577

[CR45] 45. Turon, F. *et al.* Predicting portal thrombosis in cirrhosis: A prospective study of clinical, ultrasonographic and hemostatic factors. *J Hepatol* **75**, 1367–1376, doi:10.1016/j.jhep.2021.07.020 (2021). 10.1016/j.jhep.2021.07.020 34333101

[CR46] 46. Kamat, M. A. *et al.* PhenoScanner V2: an expanded tool for searching human genotype-phenotype associations. *Bioinformatics* **35**, 4851–4853, doi:10.1093/bioinformatics/btz469 (2019). 10.1093/bioinformatics/btz469 PMC6853652 31233103

